# Draft genome sequence of *Neofusicoccum caryigenum* associated with pecan leaf dieback

**DOI:** 10.1128/mra.00944-23

**Published:** 2024-03-14

**Authors:** Erin Arthur, Young-Ki Jo

**Affiliations:** 1 Department of Plant Pathology and Microbiology, Texas A&M University, College Station, Texas, USA; University of California Riverside, Riverside, California, USA

**Keywords:** pecan leaf dieback, *Neofusicoccum caryigenum*, genome

## Abstract

Pecan leaf dieback caused by *Neofusicoccum caryigenum* is an emerging disease in southeastern United States pecan orchards. In this study, a first draft *N. caryigenum* genome was sequenced and assembled. Genome size was estimated as 42.5 Mbp, and genome completeness was estimated as 97.4%.

## ANNOUNCEMENT

*Neofusicoccum caryigenum* was first identified in 2021 as the causal pathogen of the emerging disease, pecan leaf dieback (1). This disease causes defoliation of pecan trees which can lead to decreased pecan yields. Pecan leaf dieback symptoms have been observed in the southeastern U.S. including orchards in Georgia, Louisiana, Alabama, and Texas (2–5). In this study, the genome of *N. caryigenum* was sequenced, assembled, and annotated for the first time.

The fungal culture (R1) was isolated from a pecan tree that showed symptoms of pecan leaf dieback in Texas in October 2021. Plant tissue showing necrosis on rachises was cut, surface disinfected with 10% bleach, washed with sterile water, and plated on potato dextrose agar (PDA) media with 10 µg/ml ampicillin. Subsequent hyphal-tip transfers were done onto PDA with ampicillin, and the pure culture was identified by morphology and sequencing of internal transcribed spacer (ITS) region using primers (ITS1 and ITS4) (GenBank: OR768994.1). For long-term storage, mycelium on PDA was stored in a 4-mL clear glass screw cap vial containing 1.5 mL of sterile distilled water in 15°C–25°C. *N. caryigenum* isolates are available upon request to the corresponding author. The isolate (R1) was grown out on two PDA plates with ampicillin for 1 week. The aerial mycelia were collected from both plates, flash frozen with liquid nitrogen, and ground with a mortar and pestle. DNA extraction from the mycelia was conducted using ZymoBIOMICS DNA Miniprep Kit (Irvine, CA, USA). The DNA sample was sent to Novogene Corporation Inc. (Sacramento, CA, USA) for microbial whole-genome sequencing. The DNA was randomly sheared into fragments of about 350 bp. A whole-genome paired-end library was prepared using NEBNext Ultra II DNA Prep Kits and sequenced using Illumina Novaseq PE150 technology (Illumina, San Diego, CA, USA).

Sequence data processes and analyses were performed on Texas A&M University’s High-Performance Research Computer Grace cluster. Filtering of raw reads (37,053,464 bp) and removal of adaptors were done using Trimmomatic/0.39-Java-11 (6) with the following settings: leading and trailing set to 20, sliding window of 4:20, and a minimum length of 36. Bacterial contamination was identified using Barrnap program (version 0.9) (7) through prediction of 5S, 16S, and 23S rRNA bacterial genes. The bacterial contamination was output into a file and removed by using the filter sequences by ID (8) program within the Galaxy platform (v23.0) as described by Thomas et al. (9). The cleaned reads were assembled using the *de novo* assembler, Megahit (version 1.2.9) (10), in Galaxy using Kmer sizes of 21, 29, 39, 59, 79, 99, 119, and 141 and the minimum length of contigs of 200. Cap3 (version 10.2011) (11) in Galaxy was used to scaffold the resulting contigs with parameters of overlap length cutoff of 40 and overlap percent identity cutoff of 90. The final scaffolded assembly was quality checked and visualized using Quast (version 5.2.0) (12) (Table 1). The total genome length was 42.5 Mbp, and the GC content was 56.51% (Assembly accession: ASM3196795v1; 44 Mbp long; a GC content of 56.5%), which were similar to other *Neofusicoccum* species genome assemblies (13).

**TABLE 1:** Summary of genome statistics of *Neofusicoccum caryigenum* and other closely related *Neofusicoccum* species^*a*^

	*N. caryigenum*	*N. umdonicola*	*N. ribis*	*N. parvum*
Number of contigs (>0 bp)	609	2,424	783	18
Total length of genome assembly (Mbp)	42.5	42.3	43.3	44
GC content ratio (%)	56.61	57	56.5	57
N50 (kbp)	22.9	36.5	155.9	2,500
L50	53	345	74	7
Genbank accession	ASM3196795v1	ASM982936v1	ASM2143692v1	ASM982936v1

^*a*^ The assemblies of *N. ribis* and *N. umdonicola* are selected because of the most closely related species to *N. caryigenum*. *N. parvum* has the most sequenced genomes in the *Neofusicoccum* genus. N50 is the sequence length at which 50% of the genome is contained in equal or longer sequences in kilobase pair. L50 is the minimum number of contigs that represent 50% of the genome.

## Data Availability

The *N. caryigenum* draft genome assembly has been deposited at GenBank under the accession number ASM3196795v1.
